# Retracted: How to Perfuse: Concepts of Cerebral Protection during Arch Replacement

**DOI:** 10.1155/2016/7147584

**Published:** 2016-04-07

**Authors:** BioMed Research International

The article titled “How to Perfuse: Concepts of Cerebral Protection during Arch Replacement” [1] has been retracted as it was found to contain passages of text copied from the following published article: “Modern temperature management in aortic arch surgery: the dilemma of moderate hypothermia,” by Maximilian Luehra, Jean Bachetb, Friedrich-Wilhelm Mohra, and Christian D. Etza, in The European Journal of Cardio-Thoracic Surgery, vol.45, no. 1, pp.27–39, 2014. Dr. Andreas Habertheuer has assumed all responsibility for the copied text in the article.
